# The Impact of Introducing Successive Biosimilars on Changes in Prices of Adalimumab, Infliximab, and Trastuzumab—Polish Experiences

**DOI:** 10.3390/ijerph18136952

**Published:** 2021-06-29

**Authors:** Olga Barszczewska, Anna Piechota

**Affiliations:** 1 Department of Management and Logistics in Healthcare, Medical University of Lodz, 90-131 Lodz, Poland; 2 Department of Insurance, University of Lodz, 90-001 Lodz, Poland; anna.piechota@eksoc.uni.lodz.pl

**Keywords:** biosimilar, health policy, reimbursement, health expenditure, public health, adalimumab, infliximab, trastuzumab

## Abstract

Biosimilars are cheaper than original drugs and are thus of interest to the public. The aim of this article is to assess the benefits of introducing more than one biosimilar for the same substance (active pharmaceutical ingredient, API). The hypothesis is that the introduction of successive biosimilars of a specific original drug reduces the price of the selected API. The study focuses on drug prices varying with the successive arrival of new biosimilars. Three drugs that have at least three reimbursed biosimilars on the market were selected, two from the same therapeutic group (adalimumab and infliximab) and one (trastuzumab) representing another class of drugs. The following data were analyzed: price variation after the introduction of the first, second, and third biosimilar, and the average price reduction for all three biosimilars. Additionally, a literature review was conducted. The reimbursement of each new biosimilar is beneficial since it is associated with a price reduction in percentage terms. However, the first biosimilar brought about the greatest savings due to the higher initial prices of the original drugs and to Polish reimbursement rules. This article is helpful for when taking healthcare decisions regarding the pricing of and reimbursement for new biosimilars.

## 1. Introduction

Biosimilars are biological medicinal products that are highly similar to other, already registered original medicines (“reference medicines”) [1] that are used in the treatment of patients with cancers, and infectious, autoimmune, neurodegenerative, and rare diseases [2].

In Poland, original biologic drugs and biosimilars are both most often used within drug programs, i.e., benefits included in the package of state-funded guaranteed benefits [3]. A drug program is a health reimbursement scheme including a drug technology or foodstuff for special nutrition where an active ingredient for a given indication and for a specific population is not a cost component of other guaranteed benefits [4]. Drug programs are developed by the Ministry of Health, and they are implemented, carried out, financed, monitored, and supervised by the National Health Fund (the public payer) [5]. Treatment is provided for selected diseases and includes a strictly defined group of patients. The descriptions of the programs include: patient selection criteria (e.g., disease name, comorbidities, age, and previous therapies), exclusion criteria from the program, dosing schedule, and list of required diagnostic tests. Such a detailed description significantly narrows the patient population [6]. Patients covered by the drug program receive free-of-charge treatment, and the decision on patient qualification is made by a doctor of a healthcare institution that has a contract with the National Health Fund for a specific drug program. In 2018, in Poland, there were 92 different drug programs in which 131,000 patients participated [7]. About two-thirds of the Polish programs cover nononcological treatments, and one-third covers oncological ones [8]. The drugs selected for this study, namely, adalimumamb, infliximab and trastuzumab, are reimbursed within such drug programs. Other examples of medicines reimbursed in this way are those included in programs together with adalimumab and infliximab, e.g., certolizumab, etanercept, golimumab, rituximab, tocilizumab, ustekinumab, and vedolizumab [9].

Drug programs provide patients with healthcare using innovative therapies that would be beyond the financial reach of the average individual. Such therapies are more expensive than those financed within open reimbursement or chemotherapy [10] programs, and their realization is dependent on the budget that the public payer has at its disposal [11]. Between 2016 and 2020, the total amount that the public payer spent for the reimbursement of medicines within drug programs was PLN 17.62 billion (EUR 3.96 billion), with the yearly cost increasing each year [12]. Expenditure on drug reimbursement within drug programs grows more quickly than the expenditure on open reimbursement and chemotherapy does; over 2012–2019, it increased by over PLN 2 billion (EUR 0.45 billion), i.e., by 100% [13].

The introduction of biosimilars to the market, which are less expensive than their reference medicines are, gives the tax payer a chance for drug programs cost reduction. In 2011, the expected level of price difference between a biosimilar and its relevant original equivalent was 15%–30% [14]. The production and sales of biosimilars are possible since patents for original drugs expire after 10 years [15].

The European Union was the first to establish a regulatory pathway for biosimilar registration, which is initiated by obtaining approval from the European Medicine Agency (EMA). The first biosimilar in the EU was registered in 2005. In 2018, there were already 43 registered biosimilars [16]; in March 2021, this number was 67 [17]. In 2009, a shortened pathway for biosimilar registration and substitution regulations was introduced in the USA (The Biologics Price Competition and Innovation Act of 2009, BPCIA). The first biosimilar was registered in 2015; in 2018, there were nine biosimilar medicines registered [16].

Marketing authorizations for biosimilars are required in all EU member states; however, detailed regulations of biologic drug substitution are individually imposed by each member state [18,19]. With the introduction of biosimilars, it was possible to achieve a significant level of savings. In 2016–2020, savings in the five leading EU healthcare markets amounted to over EUR 10 billion [18,20].

Regulations governing the introduction of biosimilars in specific countries are different. The applied policy in Poland is based on a simplified procedure of providing reimbursement and establishing an official selling price. The official selling price needs to be set at a level not exceeding 75% of the official selling price of the only substitute reimbursed within a given indication. Another rule is the automatic setting of a financing limit at the level of the wholesale price of the daily drug dose (DDD) of the first substitute reimbursed within a given indication. If successive substitutes are covered by reimbursement, the rule is that the limit basis in this limit group cannot be higher than the wholesale price of a daily drug dose of the first substitute [21,22]. The introduction of the first drug and of successive biosimilars should also reduce the price of medicines through creating competitive conditions between drug manufacturers. As a consequence, it should cut the costs of drug programs, improve the effectiveness of funds spent by the public payer, and increase the number of patients participating in drug programs.

The above considerations impose the question of how introducing biosimilars (the first and successive ones) to reimbursement lists might affect the price of the original drugs. The authors formulated a hypothesis that the arrival of successive biosimilars reduces the price of 1 mg of the original drug active ingredient. Additionally, the authors attempted to identify the price change of a specific active ingredient on the basis of the reimbursement-list prices in the case of original drugs with more than three biosimilars.

## 2. Materials and Methods

### 2.1. Phase 1: Medicine Selection 

In order to select the original medicines and biosimilars to be included in the study, the following criteria were applied:

- The original drug should have been approved for marketing in the European Union.
- The original drug should have at least three biosimilars approved for marketing in the European Union.
- The original drug and its biosimilars should be covered by drug-program reimbursement.

The reimbursement list was verified, and drugs fulfilling the aforementioned criteria were selected in March 2020. The first two requirements were met by five active ingredients: adalimumab, filgrastim, infliximab, pegfilgrastim, and trastuzumab. The third criterion, i.e., reimbursement under drug programs, was met by three medicines, i.e., adalimumab, infliximab, and trastuzumab. Filgrastim is available within open reimbursement and medicines applied in chemotherapy, whereas pegfilgrastim is only reimbursed within chemotherapy. The original drugs and biosimilars meeting the criteria are presented in Table 1.

Adalimumab, infliximab, and trastuzumab are biological drugs representing the group of monoclonal antibodies. A monoclonal antibody is a type of protein that detects and binds to specific structures (antigens) that exist on certain body cells. Adalimumab and infliximab were designed to detect tumor necrosis factor (TNF). The substance is involved in the process of inflammation development. Adalimumab and infliximab inhibit the effects of TNF, thereby reducing inflammation and other symptoms of diseases. Adalimumab and infliximab are used in dermatology, rheumatology, and gastroenterology [23,24]. They are covered by reimbursement in the treatment of psoriasis, psoriatic arthritis, rheumatoid arthritis, ankylosing spondylitis, Crohn’s disease, ulcerative colitis (infliximab only), and uveitis (adalimumab only). Trastuzumab binds to the HER2 receptor, of which the overexpression occurs in approximately one-fourth of breast-cancer cases and in one-fifth of gastric-cancer cases. Due to its action, trastuzumab is reimbursed in the treatment of breast and advanced gastric cancers.

### 2.2. Phase 2: Identification of Price-Level Changes

In order to identify the price changes within the selected substances, data from 47 reimbursement lists (including wholesale prices, doses per package, date of introduction of the drug to the reimbursement list) were analyzed [9]. The analyzed reimbursement lists were published between July 2012 and March 2020. The assumed initial date of publication corresponds to the date in which the original drug was entered in the reimbursement list.

### 2.3. Phase 3: Analysis of Price Change Levels

On the basis of the analysis of reimbursement lists, changes in the prices of the selected active ingredients that were related to the introduction of specific biosimilars to the reimbursement list were identified. For the identified changes in price, the wholesale gross price of 1 mg was calculated. To avoid any confusion related to packages, dosage, or formulation, the authors calculated the price per 1 mg of the active pharmaceutical ingredient. Therefore, packaging details were omitted to make the data more comprehensible to the reader. Further analysis included the following elements:

- price change in reference to basic observation (single-base indices, 100 = July 2012);
- price change in reference to a prior price change (single-base indices, 100 = prior price change).

Analysis of price changes was carried out according to the chronological order of the data included in the reimbursement lists. A biosimilar that appeared first on the reimbursement list was called the first. The biosimilars that followed the first were called the second, the third, and fourth biosimilars. In a few cases, two biosimilars of the same active ingredient entered reimbursement at the same time (in the same month and year). In such an event, the only biosimilar that was analyzed was the one with the lower price. For this reason, the number assigned to a biosimilar does not always correspond to its actual number on the reimbursement list.

The focus of this study was to analyze changes associated with the arrival of biosimilars. Therefore, the authors do not present price changes over the year and changes associated with other events. However, the dates of those changes are reported in Section 3, so as to be transparent that there were other price changes that were not related to the arrival of biosimilars.

## 3. Results

### 3.1. Adalimumab

In the studied period, five changes in the price of 1 mg of the active ingredient of adalimumab (original medicine brand name: Humira) were identified, i.e., in January 2013, January 2014, January 2019, March 2019, and March 2020. The last three price changes were related to the fact that other biosimilars were included in the reimbursement program, i.e., Imraldi (January 2019), Amgevita and Hyrimoz (March 2019), and Idacio (March 2020).

On the study start date (July 2012), the price of 1 mg of adalimumab’s active ingredient was PLN 55.58. The inclusion of Imraldi into the reimbursement program resulted in a reduction in the price of 1 mg of the original drug to PLN 26.28, which means that the price was decreased by 53%. The inclusion of two other biosimilars, Amgevita and Hyrimoz (Hyrimoz is less expensive than Amgevita) in the reimbursement program caused a 3% reduction in the price of 1 mg of the original drug, lowering it to PLN 25.52. The last biosimilar, Idacio, also led to a further drop in the price by PLN 5.50, which was a 22% reduction compared to the price obtained after the previous change.

Introducing the biosimilars into the reimbursement list caused a 64% reduction in the price of 1 mg of the original medicine on March 2020, which was the last datapoint that we analyzed, compared to in the start date. The biggest reduction in the price of the original drug was caused by the introduction of the first biosimilar, Imraldi, whereas the smallest decrease occurred after the introduction of Hyrimoz. Table 2 presents changes in the price of adalimumab that could be observed when successive biosimilars were introduced to the reimbursement list.

### 3.2. Infliximab

In the studied period, five changes in the price of 1 mg of the active ingredient of infliximab (original medicine brand name: Remicade) were identified, i.e., in January 2013, January 2014, July 2018, January 2019, and March 2019.Three of those price changes were related to the fact that biosimilars Remsima, Inflectra, Flixabi, and Zessly were introduced to the reimbursement list.

On the study start date (July 2012), the price of 1 mg of infliximab’s active ingredient was PLN 22.62. Introducing the first two biosimilars, Remsima and Inflectra, to the reimbursement list at the same time in January 2014 resulted in a 33% reduction in the price of 1 mg of the drug (PLN 7.32) compared to the price of the original medicine at the start date. Entering Flixabi on the reimbursement list (January 2018) did not bring any price drop. However, a 30% reduction in the price of 1 mg of infliximab was observed 6 months later in relation to a change in the limit basis (July 2018) to Flixabi. Introducing the last biosimilar, Zessly, to the reimbursement list resulted in a reduction in the price of 1 mg of infliximab’s active ingredient by another 18% (by PLN 1.87).

Introducing biosimilars to the reimbursement list resulted in a 62% reduction in the price of 1 mg of the original drug at the study end date as compared to the study start date. The biggest reduction (by 33%, i.e., PLN 7.32) in the price of the original medicine was caused by the introduction of the first two biosimilars, Remsima and Inflectra, whereas the smallest reduction (by 18%, PLN 1.87) occurred after the introduction of the fourth biosimilar, Zessly.

Table 3 presents observed changes in the price of infliximab when successive biosimilars were introduced to the reimbursement list.

### 3.3. Trastuzumab

In the studied period, nine changes in the price of 1 mg of the active ingredient of trastuzumab (original medicine brand name: Herceptin) were identified in January 2013, January 2014, July 2014, July 2016, July 2018, March 2019, July 2019, September 2019, and March 2020. Five of these price changes were related to the introduction of five biosimilars to the reimbursement list: Kanjinti, Herzuma, Ontruzant, Ogivri, and Trazimera. The four other price reductions were related to events other than biosimilar reimbursement, e.g., the arrival of a new drug dose.

On the study start date (July 2012), the price of 1 mg of trastuzumab’s active ingredient was PLN 19.26. Introducing the first biosimilar, Kanjinti, to the reimbursement list in July 2018 caused a reduction in the price of 1 mg of the original drug by PLN 0.64. Another price reduction in March 2019 was related to the introduction of two new biosimilars, Herzuma and Ontruzant, to the reimbursement program. Herzuma, which is less expensive than Ontruzant, was the basis for calculations and lowered the price by PLN 0.93. Introducing the fourth biosimilar, Ogivri, to the reimbursement list in July 2019 resulted in a price drop by PLN 0.98, i.e., a 9% reduction compared to the reimbursement list of the previous period. Introducing the fifth biosimilar (Trazimera) to the reimbursement list in September 2019 caused a reduction in the price of 1 mg of trastuzumab by another PLN 1.52 or 15% compared to the reimbursement list of the previous period. The reimbursement of a new dose of the Ogivri biosimilar decreased the price of 1 mg by 9%.

Introducing the aforementioned biosimilars to the reimbursement list resulted in a 59% reduction in the price of 1 mg of the original medicine compared to the start date. The biggest reduction (15%, PLN 1.52) in the price of the medicine was caused by the introduction of the fifth biosimilar (Trazimera), whereas the smallest reduction (5%, PLN 0.64) occurred when the first biosimilar, Kanjinti, appeared. Table 4 presents changes in the price of 1 mg of trastuzumab in the analyzed period.

### 3.4. Comparative Analysis

**Figure 1:**
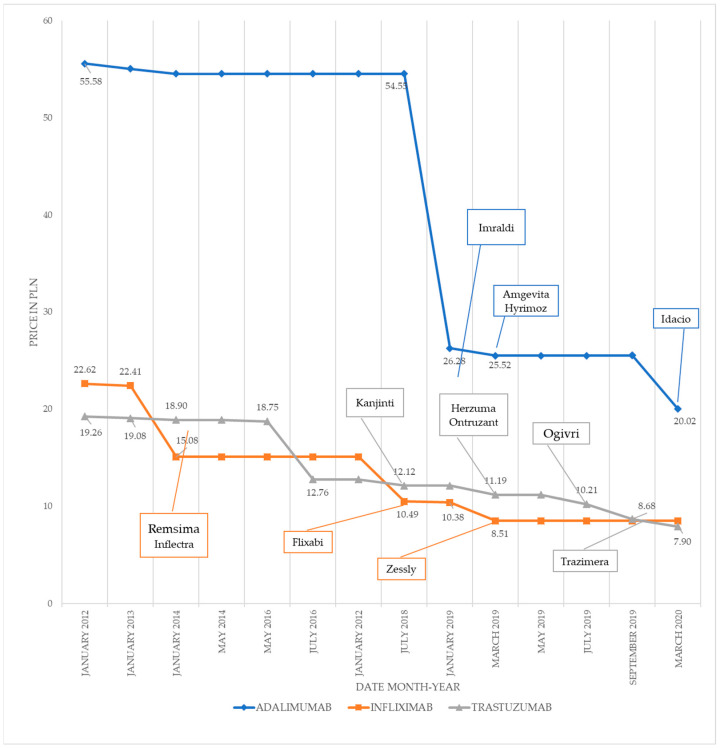
History of price changes between July 2012 and March 2020 (in PLN). Source: own elaboration based on Ministry of Health data [9].

The results of the conducted study indicate that, in the analyzed period, multifold reductions occurred in the price of 1 mg of the active ingredient in the case of all three studied medicines (Figure 1). At the end of the analyzed period, the prices of the drugs were 62% lower on average than those at the beginning of the period. The prices of adalimumab, infliximab, and trastuzumab were decreased by 64%, 62%, and 59%, respectively.

The observed reductions in the prices of specific medicines mostly resulted from the introduction of biosimilars to the reimbursement lists.

In the case of adalimumab and infliximab, the biggest price reduction was related to the introduction of the first biosimilar in the reimbursement list.

When comparing the impact of the registration of the first biosimilars with the price before their introduction, the highest reduction occurred in the case of adalimumab, whereas the smallest reduction was in the case of trastuzumab.

The largest reduction (8%) in the price of the active ingredient after the introduction of the second biosimilar was observed in the case of trastuzumab. The inclusion of the third biosimilar brought the highest percentage of price reduction in the case of adalimumab.

No similar price reductions were observed when biosimilars were grouped according to the sequence in which they were introduced. For biosimilars that were introduced first, price reductions fluctuated between 3% (infliximab) and 52% (adalimumab). In the group of biosimilars that were introduced second, the reduction range was less wide, from 0% to 8%. The third biosimilars reduced the price by 9% for trastuzumab and 22% for adalimumab.

**Figure 2:**
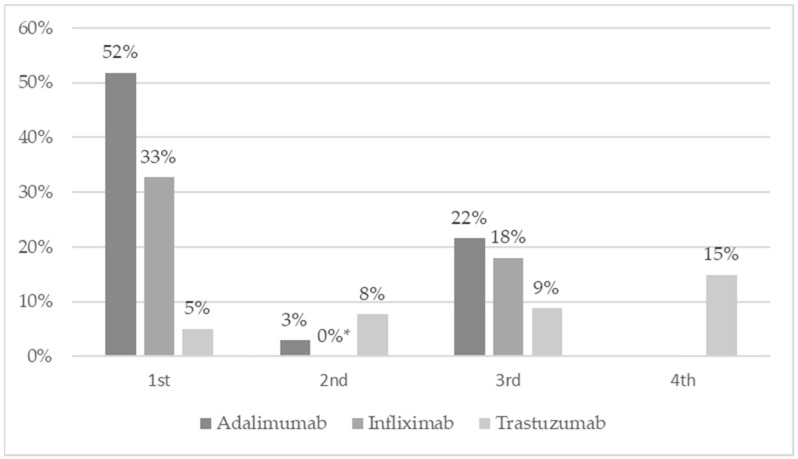
Comparison of price drops correlated with the introduction of consecutive biosimilars in percentage terms (current price vs price from the previous reimbursement list). Source: own elaboration based on Ministry of Health data [9].

The price reductions that occurred after the introduction of successive biosimilars (current price vs price from the previous reimbursement list) are shown in Figure 2.

## 4. Discussion

In the face of growing financial limitations and the expiration of patents protecting biologics, biosimilars are a group of products with a great future [26]. The loss of patent protection for many biologics could lead to the introduction of many biosimilars in the coming years [18]. The biosimilar market is regarded to be one of the most rapidly developing branches of the global pharmaceutical market. In 2017, the global market for biologics reached EUR 238 billion. The European market accounts for approximately 90% of the global sale of biosimilars. The average annual growth rate (AAGR) of biosimilars in 2016–2021 was predicted to be 53.7%, and sales in the USA were predicted to reach EUR 36.6 billion in 2021 [14,27]. However, despite the growing expansion of biosimilar markets, their share in the treatment of specific diseases still remains low. Analysis conducted in 2015 showed that the penetration of biosimilar drugs ranged from 7% in Greece to 81% in Germany; in Poland, it was 55% [18].

In the opinion of researchers, differences in the level of biosimilar market share depend on a combination of many variables, such as launch price, reimbursement system, the establishment of medicine prices in particular countries and their economic situation [28], and domestic policies of drug substitution.

Although the applied reimbursement procedure for biosimilars is simplified, the most important step remains price negotiations. This means that the reimbursement process may end up with a refusal if an acceptable price for the public payer is not reached. Official sale prices agreed upon by the Ministry of Health with manufacturers are maximal prices [18]. Substances showing similar therapeutic activity and a comparable mechanism of action are categorized in the same limit groups. Each of these groups has a certain budget ceiling, i.e., an assigned maximal cost that the National Health Fund pays for a given drug. Original medicines and their biosimilars are reimbursed within the same limit group, which causes further price reductions [29]. The process of establishing an official sale price, including the price of the first biosimilar drug, the mechanism of setting the financing budget in a budget group, and copayment amounts are tools applied by the payer that help control drug-reimbursement funds [30]. The amount of public funds that the public payer has at its disposal are strictly defined and thus limited. In Poland, the overall budget for drug reimbursement does not exceed 17% of the total amount of public funds allocated for financing guaranteed benefits within the National Health Fund [31]. By applying the available tools, the public payer attempts to effectively use the funds that are becoming increasingly smaller each year according to some sources [32].

Maximal prices define the maximum at which medicines are purchased by hospitals. When buying a drug, a hospital needs to allocate a budget for this purpose, which is estimated on the basis of data from reimbursement lists and available funds. Medicines are purchased by hospitals in compliance with the public procurement procedure [33]. Over the course of this procedure, manufacturers submit tender offers out of which a hospital chooses the most favorable one in accordance with the adopted criteria. The purchase criterion is most often only the offered price. A hospital chooses the least expensive offer, which results in competitive conditions between drug distributors. They must offer medicines to hospitals at equal or lower prices (a drug may not be sold at a higher price) than the official sale prices. The applied purchase procedure may result in the drug sale price being lower than the maximal price. Thus, it is often the case that market competition for hospital sales has stronger impact on price reduction than that of initial market-access negotiations [34].

Some countries within the European Economic Area, e.g., Norway, apply public procurement procedures held by one payer in the case of medicines administered in hospital. As a result of aggressive negotiations, the payer reaches prices that are 70% lower (as in the case of a biosimilar of infliximab). On the other hand, in the United States, where the payer and pharmacy managers conduct undisclosed negotiations, the price of a biosimilar of infliximab was only 22% lower than that of the original drug. Sometimes, a competitor reduces the price of the original drug itself. The price of the original drug is often reduced when a competing product enters the market. For example, in Sweden, AbbVie lowered the price of the original adalimumab by 80% [35].

Due to the system of international reference pricing being in force in CEE and CIS countries, the prices of new drugs are often adjusted to those applied in countries with higher income. When comparing the costs of health benefits in poorer countries, such a price may not be justified by healthcare-system savings resulting from the avoidance of medical events or hospitalization. For this reason, innovative drugs are not cost-effective in these countries. Additionally, less prosperous countries might not allocate as high a budget for the purpose of decreasing morbidity or mortality rates. Therefore, many countries, especially those with a lower income, apply tacit discounts, confidential price agreements, and other cost limitations. Other used methods are based on restrictions of access to medicines, e.g., by delaying reimbursement, limiting reimbursed indications with regard to the summary of product characteristics, restricting the number of patients, or shortening therapy duration [36].

When establishing a drug policy, attention should be paid to the potential long-term effects of price reduction, e.g., decreased interest of manufacturers and competition impediment [18]. However, regardless of savings, the main grounds for taking a therapeutic decision should be clinical factors, and the safety and quality profiles of the drugs [35].

A policy regulating the switch of original drugs with biosimilars is not specified by the EMA [34]. Therefore, each country independently chooses its strategy. In Poland, Lithuania, and Slovakia, substitution is partly obligatory due to the selection of medicines that win a hospital tender procedure. In countries where tenders are not conclusive, decisions are made by doctors. Some countries, e.g., Hungary, introduced incentives for doctors who prescribe biosimilars to at least 40% of their existing patients. In Belgium, biosimilars must be used by at least 20% of new patients [36].

The Polish reimbursement list of the Ministry of Health, adopted as the basis for this analysis, was a key instrument for observing changes in the study areas. Our conducted research showed that the reimbursement list, and thus the related policy, had huge impact on the chances of reaching the savings planned by the public payer.

In the case of adalimumab, the introduction of the four biosimilars to the market contributed to a reduction in the price of 1 mg of the substance by 64%. This is consistent with data from another Polish report in which, in December 2018, the price of adalimumab was reported to be reduced by 53% compared to the price of the original drug [18]. Additional data indicate that, in the middle of 2019, biosimilars constituted only about 5% of the market share within the adalimumab market [18]. In the case of infliximab, the arrival of the four biosimilars on the market contributed to a price reduction in the 1 mg of the substance by 62%, which was also confirmed by the results of other studies [18]. The market share of infliximab in the middle of 2019 was approximately 95% [18], from 87.5% up to 99.9% [37]. In the case of trastuzumab, the introduction of the five biosimilars in the market contributed to a 59% reduction in the price of 1 mg of the substance. This also corresponds to data found in other studies. The market share of trastuzumab was approximately 25% of the substance in the middle of 2019 [18].

Including successive biosimilars in the reimbursement program, and the increased market share of biosimilars do not necessarily translate into an increase in the number of patients receiving treatment. This phenomenon was observed in rheumatology, where it is estimated that only 1.3%–2.94% of patients in Poland suffering from rheumatoid arthritis [34] underwent biological drug treatment, which is a very low percentage rate compared to that in other EU member states.

In other countries, higher reductions in the price of biosimilars compared to the reference drug were also observed. In Norway, a reduction in the price of an infliximab biosimilar reached 69% in 2015, whereas it was 60% in 2016; in Great Britain, it was 59% in the period from March 2016 to February 2017 [34].

In the summary published by Serwis Pharma Compass in 2016, as many as four original biologics in rheumatology, gastroenterology, and dermatology, among others, were among the top five drugs with the world’s highest sales turnover. The first drug was adalimumab, which was analyzed in this article, and the third to fifth positions were taken by etanercept, infliximab, and rituximab, respectively [34,38].

In the United States, the annual growth rate of the biosimilar market share in 2016–2021 was predicted to be 54%, whereas sales in 2021 were predicted to be EUR 36.6 billion [18,27]. These estimates show that a 20% reduction in the prices of six nonpatented drugs may result in savings reaching EUR 1.6 billion. As for rituximab, it is estimated that the substitution of the original drug with a biosimilar in the treatment of non-Hodgkin’s lymphoma and chronic lymphocytic leukemia in Europe may bring annual savings amounting to EUR 56.82 million [39].

The European experiences in specific substances appear to confirm the abovementioned prognosis, both with regard to the expected savings and to an increase in the number of treated patients. For erythropoietin, a reduction in the price by 25%–30% and the substitution of the original medicine with biosimilars resulted in savings that provided additional therapy with rituximab (9770 therapies), bevacizumab (3912 therapies), and trastuzumab (3713 therapies). Regarding rituximab, etanercept, and infliximab, it was estimated that substituting the original drugs with their biosimilars brought the British payer savings amounting to GBP 210 million in 2017–2018 [39]. The experiences of the Polish payer in this respect show that, in 2014–2019, the introduction of an infliximab biosimilar provided savings of approximately PLN 168 million; in the case of etanercept, the amount was about PLN 141 million [18].

Apart from savings obtained directly from used biosimilars instead of the original medicines, biosimilar drugs may offer other advantages.

One of the most interesting strategies is a scheme applied in several Italian regions. According to this strategy, the payer is obliged to allocate half of the savings to increasing the budget for innovative drugs by 20%, and for extending reimbursement indications within already existing therapies [18]. Another advantage in the context of both specific patients and society as a whole is preventing the loss of resources due to a disease and its consequences. Such resources may include productivity and capacity for work among patients and their family members or friends who take care of them. This translates into gained benefits by both specific patients and by society (e.g., with regard to the gross national product) [18,40,41].

Due to a number of factors, it is difficult to fully use the potential of biosimilars. Inotai et al. (2018) [36] identified the main barriers for biological therapies in the Central and Eastern European countries (CEE) and Commonwealth of Independent States (CIS) to be the limited number of patients who may undergo treatment financed with public funds, queues, and a limited therapy period. Other mentioned obstacles were the lack of reimbursement for relevant diagnostic procedures, additional therapy qualification criteria, and limited reimbursement indications [36]. A 2019 report [18] also emphasized the negative impact of very restrictive drug-program access to biological therapies. These restrictions are imposed to secure the payer’s budget; however, they often deviate from Polish and European recommendations given by scientific societies. Moreover, Poland is the only country in Europe that applies provisions under which an effective therapy is discontinued after a period specified in the program, and restarted only in the case of patient relapse. Another obstacle is the low pricing of health benefits (as a service) in drug programs with a simultaneous increase in the number of duties that a doctor or a healthcare unit must fulfill, which discourages hospitals from providing certain medical services. Due to a lack of financial benefits for hospitals, in most cases, hospital staff members do not receive any extra remuneration [18].

This article assesses the benefits of introducing more than one biosimilar for the same substance (active pharmaceutical ingredient, API). Therefore, extensive data from July 2012 to March 2020 were analyzed. To present the broader context, a literature review was conducted, and results were presented in the discussion. The presented approach sheds new light on possible savings that biosimilars might bring. This could be useful for decisionmakers when creating health-policy strategies. It is a particularly important topic, taking into consideration the limited healthcare budgets of each country.

The limitations of this study include the fact that the data concern only three drugs, and the study was exclusively based on Polish data. In addition, the pricing process is very complex and influenced by many factors, and the reimbursement of biosimilars is only one of them. It is interesting to explore if the price of second biosimilars is influenced by that of third biosimilars. Further analyses based on more drugs and an international context would be interesting.

The use of biosimilars also poses many challenges, such as immunogenicity risk, substitution rules, spreading information about biosimilars and social awareness of them, acceptance in the medical community, required clinical research prior to market introduction, an expensive drug registration pathway that potentially reduces financial gains, and political barriers [39].

## 5. Conclusions

According to Polish reimbursement principles, biosimilars introduced to reimbursement lists must be cheaper than their original drugs. This triggers two effects. First, the use of biosimilars within specific drug therapies creates conditions for competitiveness between original medicines and biosimilars. Second, introducing a biosimilar into a reimbursement list results in a reduction in the original drug’s price.

There is no rule as to which biosimilar entering a reimbursement list would cause the largest price reduction. However, each successive biosimilar introduced to the reimbursement list entails another drop in the price of the original drug.

Ultimately, the obtained reduction may enable the public payer to achieve the expected cost savings with regard to particular drug programs, even if the original drug is still used in therapy. These savings are crucial since the public payer is incurring full expenditure.

The implementation of statutory law on the use of savings achieved this way should specify that they may only be allocated for providing treatment to a larger number of patients within the same disease or another therapeutic area. This solution provides more options for the widespread use of both original drugs and biosimilars.

## Figures and Tables

**Table 1:** Drugs selected for this study.

Substance	Original Drug	First Biosimilar	Second Biosimilar	Third Biosimilar	Fourth Biosimilar	Fifth Biosimilar
Adalimumab	Humira	Imraldi	Amgevita Hyrimoz	Idacio	x	x
Infliximab	Remicade	Remsima Inflectra	Flixabi	Zessly	x	x
Trastuzumab	Herceptin	Kanjinti	Herzuma Ontruzant	Ogivri	Trazimera	New dose Ogivri

Source: Ministry of Health data [9].

**Table 2:** Gross wholesale price changes between July 2012 and March 2020 of adalimumab after biosimilar reimbursement.

Drug		Humira	Imraldi	Amgevita and Hyrimoz	Idacio
Event		Original Drug	First Introduction of Biosimilars	Second Introduction of Biosimilars	Third Introduction of Biosimilars
Date		July 2012	January 2019	March 2019	March 2020
Price per package		4446.75	2102.67	2041.2	800.66
Price per 1 mg		55.58	26.28	25.52	20.02
Current price vs. first price from July 2012	PLN	x	−29.30	−30.07	−35.57
	%	x	−53%	−54%s	−64%
Current price vs price from previous reimbursement list	PLN	x	x	−0.77	−5.50
	%	x	x	−3%	−22%

Middle exchange rate: EUR 1 = PLN 4.45 [25]; source: own elaboration based on Ministry of Health data [9].

**Table 3:** Gross wholesale price changes between July 2012 and March 2020 of inliximab after biosimilar reimbursement.

Drug		Remicade	Remsima and Inflectra	Flixabi		Zessly
Event		Original Drug	First Introduction of Biosimilars	Second Introduction of Biosimilars	Becomes Limit Basis—Price Drop	Third Introduction of Biosimilars
Date		July 2012	January 2014	January 2018	July 2018	March 2019
Price per package		2261.77	1508.22	1508.22	1048.95	850.50
Price per 1 mg		22.62	15.08	15.08	10.4895	8.51
Current price vs first price from July 2012	PLN	x	−7.54	−0.00	−12.13	−14.11
	%	x	−33%	−33%	−54%	−62%
Current price vs price from the previous reimbursement list	PLN	x	x	0.00	−4.59	−1.87
	%	x	x	−0% *	−30%	−18%

Middle exchange rate: EUR 1 = PLN 4.45 [25]; * 0% change in the price in January 2018, when it was introduced. Second biosimilar reduced price by 30% when it became the limit basis in July 2018. Source: own elaboration based on Ministry of Health data [9].

**Table 4:** Gross wholesale price changes between July 2012 and March 2020 of trastuzumab after biosimilar reimbursement.

Drug		Herceptin	Kanjinti (2 Doses)	Herzuma and Ontruzant	Ogivri	Trazimera	Ogivri
Event		Original Drug	First Introduction of Biosimilars	Second Instroduction of Biosimilars	Third Instroduction of Biosimilars	Fourth Instroduction of Biosimilars	New Dose
Date		July 2012	July 2018	March 2019	July 2019	September 2019	March 20
Price per package (PLN)		2889	7271.78	1678.32	1530.9	1302.74	3319.22
Price per 1 mg (PLN)		19.26	12.12	11.19	10.21	8.68	7.9
Current price vs first price from July 2012	PLN	x	−7.14	−8.07	−9.05	−10.58	−11.36
	%	x	−37.07%	−41.91%	−47.01%	−54.91%	−59%
Current price vs price from the previous reimbursement list	PLN	x	−0.64	−0.93	−0.98	−1.52	−0.78
	%	x	−5%	−8%	−9%	−15%	−9%

Middle exchange rate: 1 EUR = 4.45 PLN [25]; Source: own elaboration based on Ministry of Health data [9].

## Data Availability

The data presented in this study are available on request from the corresponding author.
